# Diet and Transgenerational Epigenetic Inheritance of Breast Cancer: The Role of the Paternal Germline

**DOI:** 10.3389/fnut.2020.00093

**Published:** 2020-07-15

**Authors:** Raquel Santana da Cruz, Elaine Chen, Megan Smith, Jaedus Bates, Sonia de Assis

**Affiliations:** Department of Oncology, Lombardi Comprehensive Cancer Center, Georgetown University, Washington, DC, United States

**Keywords:** breast cancer, epigenetic inheritance, transgenerational effects, diet, paternal germline

## Abstract

The past decade has made evident that in addition to passing their genetic material at conception, parents also transmit a molecular memory of past environmental experiences, including nutritional status, to their progeny through epigenetic mechanisms. In the 1990s, it was proposed that breast cancer originates *in utero*. Since then, an overwhelming number of studies in human cohorts and animal models have provided support for that hypothesis. It is becoming clear, however, that exposure in the parent generation can lead to multigenerational and transgenerational inheritance of breast cancer. Importantly, recent data from our lab and others show that pre-conception paternal diets reprogram the male germline and modulate breast cancer development in offspring. This review explores the emerging evidence for transgenerational epigenetic inheritance of breast cancer focusing on studies associated with ancestral nutritional factors or related markers such as birth weight. We also explore paternal factors and the epigenetic mechanisms of inheritance through the male germline while also reviewing the existing literature on maternal exposures in pregnancy and its effects on subsequent generations. Finally, we discuss the importance of this mode of inheritance in the context of breast cancer prevention, the challenges, and outstanding research questions in the field.

## Introduction

The genome is relatively stable throughout an organism's life span; however, the epigenome is malleable to ensure short-term adaptation to the environment (1). Because epigenetic plasticity peaks in early development, the risk of diseases such as breast cancer can result from environmental exposures acting in that life stage (2, 3). While DNA sequence is responsible for the majority of heritability of disease, including cancer, it is becoming clear that epigenetic inheritance can also occur. Indeed, environmentally induced disease risk has been experimentally shown to be transmitted from one generation to another via epigenetic mechanisms both through the female and male germlines (2, 4, 5). Although most of the evidence for this mode of disease inheritance comes from maternal exposures in pregnancy, we (6, 7) and others (8–10) have shown that paternal exposures in the pre-conception window are also important in determining disease outcomes in the offspring.

Family history is an important risk factor for breast cancer and it accounts for up to one third of all cases (11). However, mutations in high penetrance genes such as *BRCA1* and *BRCA2* explain only a small proportion of breast cancers. Despite intense search, no major genetic mutations have emerged (12). This suggests that the familial or inheritable component of some breast cancers is not only transmitted by classical genetic inheritance but could also be mediated through non-genetic mechanisms.

The majority of breast cancers are sporadic and thought to result from environmental and lifestyle exposures (13). Although a number of nutritional and lifestyle factors have been linked with breast cancer, these associations are mostly inconsistent (14). Exceptions include alcohol consumption, obesity, and inadequate physical activity, which are strongly associated with breast cancer (15–17). It has been suggested that inconsistencies between certain environmental factors and breast cancer are due to timing of exposure and windows of susceptibility at different stages of mammary gland development throughout a women's lifetime (18, 19). In line with that, it was proposed that some breast cancers may originate *in utero* (20) when the mammary tissue first arises and multiple reports on maternal exposure in pregnancy have offered support to that hypothesis (4, 21–23). However, recent findings from our lab and others argue for a contribution of paternal pre-conception exposures to offspring's breast cancer risk in rodent models.

This review explores the evidence for epigenetic inheritance of breast cancer focusing on studies associated with ancestral nutritional factors or related markers such as birth weight. While we concentrate on epigenetic inheritance through the male germline, we also reviewed the existing literature on maternal exposures in pregnancy and its effects on subsequent generations. We also discuss the importance of this mode of inheritance in the context of breast cancer prevention, the challenges, and outstanding research questions in the field.

## Developmental Origins of Breast Cancer

### Developmental Origins of Health and Disease (DOHaD) Theory

The DOHaD hypothesis states that exposures to certain environmental factors or nutritional conditions during critical developmental windows, particularly the fetal stage, can have long-lasting impact on an individual's health (24). While *in utero*, the fetus relies heavily on maternal nutrients and signals for optimal growth and health outcome later in life. It has been proposed that the fetus receives a forecast from the mother that prepares them for the type of environment they will encounter after birth. The fetus responds to these signals with adaptations (epigenetic programming) which will help it to survive after birth (2). This developmental plasticity, which enables organisms to adapt to environmental signals during early life, can also increase the risk of developing chronic diseases when there is a mismatch between perceived environment and that which is encountered in adulthood (2). For instance, during the *in utero* period, nutritional imbalance (abundance or scarcity of food or specific nutrients) induces epigenetic changes that can increase the risk of chronic diseases such as cancer. However, the disease phenotype might only manifest itself when an individual is exposed to certain environmental signals after birth (2), such as poor diet and lack of physical activity.

### *In utero* Exposures and Breast Cancer

Disruptions in early development are linked to adult-onset breast cancer. In the 1990s, it was proposed that breast cancer can begin *in utero* in response to higher estrogen levels during pregnancy (20). Estrogen levels during gestation can be modulated by nutritional factors (25) and are positively correlated with fetal growth and birth weight (26).

Although few population studies have directly investigated the association between maternal nutrition in pregnancy and breast cancer in daughters, many others have found a link between birth weight—considered a good surrogate marker reflecting the extent and quality of intrauterine growth—and breast cancer. Most of these population-based studies found a U-shaped association between birth weight and breast cancer in adulthood (27), with both high and low birth weight reported to be positively associated with increased breast cancer risk compared with normal or average birth weight (27–32). A meta-analysis found that both high weight at birth and birth length were related to increased breast cancer risk (33). Animal studies lend support to the findings in epidemiologic cohorts and show that both high and low birth weight increase the incidence of breast cancer in rodent models (21, 34). In addition to birth weight, *in utero* exposures to environmental factors such as DDT and DES are linked to increased breast cancer risk (22, 35).

In animal models, intake of specific dietary factors during pregnancy can also modulate breast cancer development in offspring. In rats, maternal consumption of a diet rich in N-6 polyunsaturated fatty acids (N-6 PUFAs) during pregnancy increased estradiol levels and subsequent risk of mammary cancer in the female offspring (25). Another study reports that maternal protein restriction in gestation and lactation is associated with low birth weight, catch-up growth after birth, and increased breast cancer risk in offspring, particularly in the presence of a high-calorie diet in adulthood (34, 36). More recently, a study showed that both excess and deficiency of dietary zinc during gestation is associated with higher breast carcinogenesis in daughters (37).

Some *in utero* exposures can lead to breast cancer reduction in animal models. For instance, prenatal exposure to a lard-based high-fat diet during pregnancy or pregnancy and lactation significantly reduced the offspring susceptibility to mammary cancer (23). In rats, maternal diet enriched in N-3 PUFA decreased the risk of breast cancer in offspring (38). Interestingly, maternal exercise also decreases mammary tumor incidence in a rodent model (39).

In animal models, *in utero* exposures that lead to increased breast cancer development also modulate mitogenic signaling pathways in mammary tissues including IGF-1, IR, MAPK, and ER-alpha [Table 1; (21, 34, 36)].

**Table 1:** Parental exposure-induced molecular changes in offspring's mammary tissues.

**Animal model**	**Ancestral experience**	**Molecular alteration**		**References**
**INTERGENERATIONAL**				
**Maternal**				
Sprague–Dawley rats	High-fat diet	Protein levels	MAPK and ER-alpha	de Assis et al. (21)
Wistar rats	Low-protein diet	Protein levels	Insulin receptor (IR) and insulin-like growth factor-1 receptor (IGF-1R)	Fernandez-Twinn et al. (34)
Wistar rats	Low-protein diet	Gene expression	e.g., *Igf-1r, Sp1, Jak2, Cdkn1a, Cdkn1b, mmp9, Serpin1, Nfkb1, Bax, and Nme1*	Fernandez-Twinn et al. (36)
		Protein levels	IGF-1R, SP1, and mTOR	
**Paternal**				
C57BL/6 mice	Obesity-inducing diet	microRNAs	e.g., miR-296-5p, miR-874, and miR-1896	Fontelles et al. (6)
		Protein levels	MAPK, HIF-1A and downstream target, and VEGF-A	
Sprague–Dawley rats	Corn oil- and lard-based high-fat diet	microRNAs	e.g., miR-1897-5p, miR-219-1-3p, and miR-376a	Fontelles et al. (7)
		Protein levels	e.g., TGFβ, AKT, mTOR, and JNK	
C57BL/6 mice	Low-protein diet	DNA methylation	e.g., *Tuba3a, Rhox13*, and *Gnas*	da Cruz et al. (40)
		sncRNAs	e.g., miR-28a, miR-200c, miR-451a, tRNA-Gly-CCC, and tRNA-Val-TAC	
		Protein levels	CAB39, AMPK, mTOR, and its downstream targets S6 kinase and eukaryotic initiation factor (eIF) 4E-binding protein	
C57BL/6 mice	DDT	Protein levels	AMPK, mTOR, and its downstream targets S6K kinase and eukaryotic initiation factor (eIF) 4E-binding protein	da Cruz et al. (41)
**TRANSGENERATIONAL**				
Sprague–Dawley rats	High-fat or ethinyl-estradiol (EE2)-supplemented diet	Gene expression	*Dnmt1* and *Dnmt3a/b*	de Assis et al. (4)
		DNA methylation	e.g., *Pax6, Runx3, Foxe3, Gata4*, and *Vgf*	
C57BL/6NTac mice	N-6 PUFA high-fat diet	Gene expression	e.g., *Akt2, Egr3, Hes1, Id4, Jam3, Pcdhga8, Slc26a10, Tbx2, Igfbp6, Oas3a, p21*, and *Slfn1*	Nguyen et al. (42)

### Intergenerational and Transgenerational Inheritance of Breast Cancer

**Figure 1:**
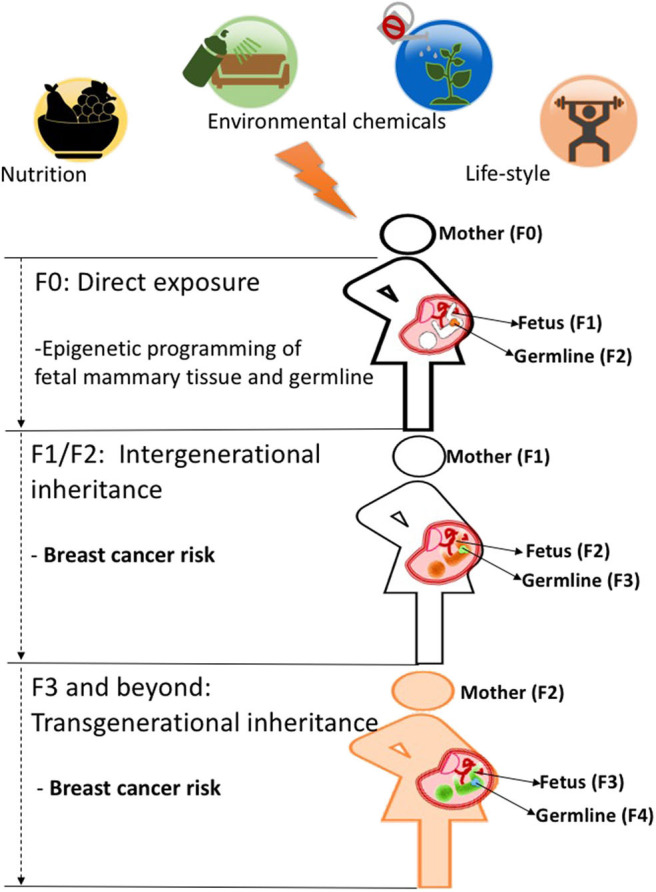
When a mother (F0, black outline) is exposed to an environmental insult such as an unhealthy diet during pregnancy, her offspring's mammary tissues (F1 generation, fetus with black outline) and its already developing germ cells (orange)-which will give rise to the F2 generation-will be directly programmed and may cause intergenerational effects on F2 generation's breast cancer predisposition (fetus with orange color). Those environmental effects on breast cancer risk can be carried to subsequent generations (F3 generation and beyond, fetus wit green color) and represent true transgenerational epigenetic inheritance as they are transmitted through the F2 germline (green) which was not directly exposed to the initial environmental insult in the F0 generation. While evidence in humans is still lacking, animal studies support the idea that transgenerational epigenetic inheritance of breast cancer predisposition occurs (4, 48, 52).

**Figure 2:**
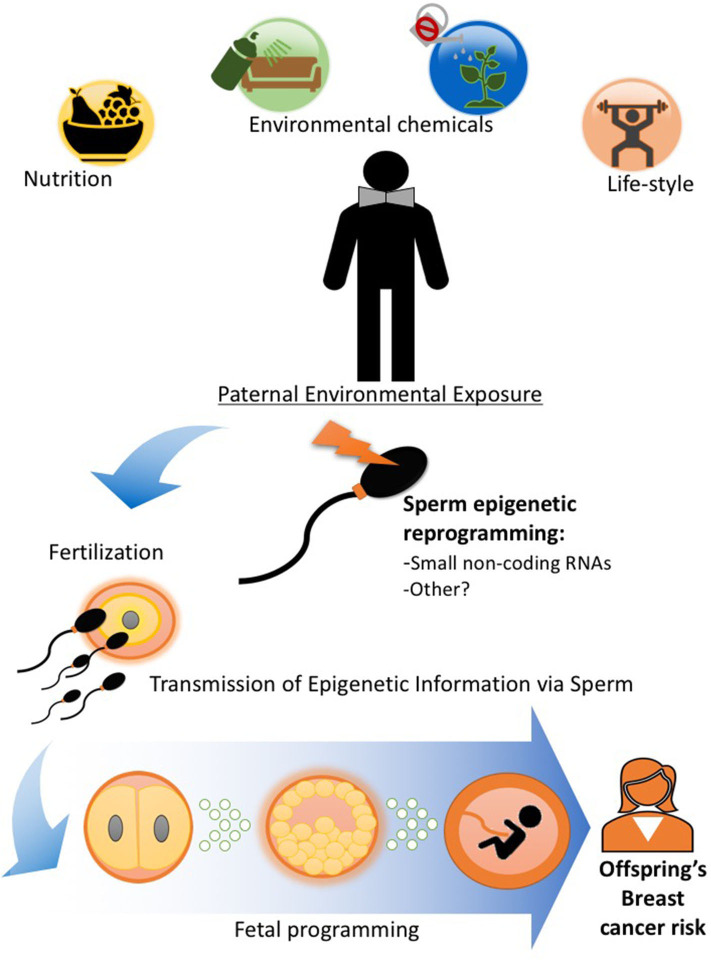
Paternal exposures and epigenetic inheritance of breast cancer. Pre-conception paternal nutritional status and exposure to other lifestyle and environmental factors can epigenetically reprogram the sperm, particularly its small non-coding RNA load. Epigenetic information in sperm can be delivered to the oocyte during fertilization (55) and impact embryonic and fetal tissue development, including that of the mammary gland and alter breast cancer predisposition in offspring. Evidence in human and animal studies support the idea that epigenetic inheritance of breast cancer predisposition can be transmitted through the male germline (6, 7, 10, 40, 41, 56–58).

As discussed above, both human and animal studies show that maternal exposures in pregnancy can increase breast cancer predisposition in offspring. This increase in cancer risk observed in offspring (F1) is likely due to fetal programming of the mammary tissue as many studies show morphological and molecular changes in that tissue induced by a number of prenatal exposures (43). However, it has been shown that *in utero* exposures can also affect developing germ cells in the F1 generation (5), which can cause intergenerational effects and impact the F2 generation's health phenotypes. Those effects can, sometimes, extend to the F3 generation and beyond. Effects observed in the F3 generation are the first evidence of environmentally induced transgenerational epigenetic inheritance as they are transmitted through the F2 germline, which was not directly exposed to initial environmental insult in the F0 generation as depicted in [Figure 1; (2, 45)]. Several studies in animal models provide examples that environmental chemicals exposure and nutritional status in early life can lead to transgenerational inheritance of phenotypes, where transmission of disease risk is passed between generations (46). Whether epigenetic marks such as DNA methylation can be directly inherited across generations via the germline is still controversial and is discussed in more detail in section Pre-conception Paternal Exposures and Epigenetic Inheritance of Breast Cancer of this review and in previous publications (47–49).

Although transgenerational effects have been reported in human cohorts (50, 51), population data for this mode of inheritance for breast and other cancers are still lacking. Still, there is experimental evidence that transgenerational inheritance of breast cancer does occur in animal models. We showed that dietary factors and endocrine disruptors can lead to transgenerational transmission of breast cancer predisposition through both the male and female germlines in carcinogen-induced rodent models of this malignancy (4, 42). In the first study, we showed that feeding pregnant rat dams isocaloric diets enriched in N-6 PUFAs (given throughout gestation) programmed increased breast cancer development in daughters (F1) and granddaughters (F2) compared to controls. This increase in breast cancer risk was transmitted to subsequent generations via both the maternal and paternal germlines, indicating that both the male and female germlines are reprogrammed by an *in utero* exposure to a N-6 PUFA diet. We also showed that maternal exposure to an ethinyl-estradiol (EE2)-supplemented diet (given from gestational days from 14 to 20) led to an increase in breast cancer predisposition not only in daughters (F1) and granddaughters (F2) but also in great-granddaughters (F3) (4). Differences in the number of generations affected with increased mammary tumorigenesis due to N-6 PUFA and EE2-supplemented diets could have resulted from the different durations of these maternal exposures. It is possible that, in order for breast cancer risk to be transmitted in a transgenerational manner, exposures need to occur within a specific window of fetal development, which allows for persistent epigenetic reprogramming of the germline, as shown for other diseases (52). DNA methylation is critical for embryonic development in mammals and DNA methylation patterns change dynamically to allow for tissue-specific differentiation (49, 53). During embryonic development, two main waves of demethylation occur, one at the zygote stage and the other when primordial germ cells reach genital ridge (49, 54). To answer whether a maternal N-6 PUFAs-enriched diet could cause transgenerational effects if given after the second wave of demethylation (which targets the fetal germline), we conducted another study where experimental diets were fed to pregnant rats between gestational days 10 and 20. Indeed, maternal N-6 PUFA consumption during this stage of fetal development induced transgenerational effects, with higher breast cancer risk observed in the F3 generation (42). The transgenerational effects on breast cancer risk were associated not only with morphological changes but also with DNA methylation and gene expression alterations in mammary tissues [Table 1; (4, 42)]. A recent rodent study supported those findings and showed that prenatal exposure to alkylphenols causes transgenerational alterations in mammary gland development and gene expression in the F3 generation females (44).

## Pre-Conception Paternal Exposures and Epigenetic Inheritance of Breast Cancer

Because of the intimate relationship between mother and the developing fetus during pregnancy, the evidence for developmental programming and epigenetic inheritance of disease comes overwhelmingly from studies investigating maternal environmental exposure during gestation and outcomes in the offspring and subsequent generations as described earlier in this review. However, a variety of pre-conception paternal experiences ranging from nutritional status, alcohol consumption, and stress to exposure to pollutants have been shown to alter the male germline epigenome and impact their children's and, sometimes, grandchildren's health [Figure 2; (8, 52, 59–61)], including cancer predisposition (6, 7, 40, 62). We (6, 7, 40) and others (10) have documented that paternal obesity and specific dietary interventions and environmental exposures in the pre-conception window play an important role in determining breast cancer in the progeny.

Using a carcinogen-induced mouse model of breast cancer, we showed that both paternal obesity and malnutrition programs development of breast cancer in offspring (6, 7, 40). Diet-induced male obesity before conception epigenetically reprogrammed the male germline, increased birth weight as well as the susceptibility of breast cancer in daughters (6). Female offspring of obese fathers had delayed mammary gland development with increased number of terminal end buds and ductal elongation (6). We and others have also investigated the effects of specific nutritional factors in paternal diet. In rats, paternal consumption of animal- or plant-based high-fat diets produced opposing effects in offspring's breast cancer predisposition, with lard-enriched diets (high in saturated fatty acids) increasing breast cancer risk in offspring while corn oil-rich diets (high in N-6 PUFAs) decreased it (7). Another study reported that pre-conception paternal selenium deficiency increased mammary carcinogenesis in female rat offspring (10). More recently, we found that daughters of fathers fed a low-protein diet (50% reduction in optimal levels) had decreased birth weight and accelerated breast tumor growth, with tumors arising earlier and growing faster than in the control group. This phenotype was associated with epigenetic modifications in normal mammary glands. Moreover, the tumor phenotype was linked to regulation of the nutrient sensing mechanisms, with suppression of AMPK and increased mTOR activity in normal mammary tissues and tumors (40). Interestingly, another study from our lab showed that oral intake of the pesticide DDT in males also led to low birth weight and mammary tissue and tumor phenotypes, which were similar to those found for paternal malnutrition, including alterations in the energy sensing AMPK/mTOR axis, suggesting that there may be a common underlying mechanism for two distinct exposures (41). Paternally induced alterations in mitogenic pathways in daughter's mammary tissues are linked to epigenetic programing in DNA methylation and small non-coding RNAs such miRNAs and tRFs [Table 1; (6, 7, 40)].

While population studies investigating paternally induced programming of breast cancer are still scarce, a few reports can be found in the literature. For instance, both paternal age and ethnicity have been associated to the risk of breast cancer in daughters (56, 57). Using the Dutch Famine Cohort, a study reported that women who were conceived during the famine (presumably with both parents being malnourished), but not those exposed later in gestation, have increased breast cancer risk (58). Another analysis in humans found an association between exposure to the endocrine-disrupting chemical dibutylphthalate in male war veterans and increased odds of breast cancer development in daughters (63). Further, epidemiologic studies have consistently shown that both high and low birth weight increases a woman's breast cancer risk (27, 28, 64). It is often assumed that birth weight is solely determined by maternal factors in pregnancy, yet population and animal studies have shown that paternal pre-conception factors also play a role in modulating birth weight in offspring (6, 40, 62, 65–67). Thus, it is likely that the reported relationship between birth weight and breast cancer risk in human cohorts reflects not only maternal factors and prenatal exposures but also pre-conception paternal exposures and needs to be further investigated in human populations.

## Mechanisms of Epigenetic Inheritance Through the Male Germline

The role of environmentally induced epigenetic reprogramming of the germline has been actively investigated in recent years (6, 8, 9, 68). While both female and male germ cells can likely be epigenetically reprogrammed by environmental insults, most studies have focused on the male germline. One reason is that the maternal germline is technically challenging to harvest due to the limited number of mature eggs produced by females during their lifetime (69). Sexually mature males, on the other hand, produce an infinite number of sperm cells in a process that continues throughout adult life (69). The second challenge when studying epigenetic programming via the female germline is the possible confounding associated with maternal exposures in pregnancy, which is known to modulate disease risk in offspring as discussed earlier. Thus, most studies investigating germline epigenetic inheritance in mammals have concentrated in male environmental exposures and its effects on sperm. In this section, we will briefly review the data associated with epigenetic reprogramming of the male germline. For a more in-depth and detailed analysis of germline epigenetic reprogramming, please refer to excellent reviews published elsewhere (55, 70).

### Epigenetic Inheritance via Sperm Small Non-coding RNAs

The proposed mechanism by which paternal environmental exposures are linked to disease phenotypes in offspring is sperm epigenetic reprogramming (Figure 2 and Table 2). While DNA methylation and histone modifications have been associated with germline transmission of phenotypes (71) from fathers to offspring, the notion that those epigenetic marks can be directly inherited across generations in mammals is still controversial given the cycles of epigenetic erasure and reprogramming occurring during embryogenesis (47–49).

**Table 2:** Summary of epigenetic and molecular alterations in male germline and in placenta linked to paternal environmental exposures.

**Animal model**	**Paternal experience**	**Epigenetic mechanism**		**References**
**SPERM EPIGENETIC ALTERATIONS ASSOCIATED WITH BREAST CANCER IN OFFSPRING**				
C57BL/6 mice	Obesity-inducing diet	miRNAs	e.g., miR-146b, miR-29a, miR-296-5p, and miR-874	Fontelles CC et al. (6)
Sprague–Dawley rats	Corn oil- or lard-based high-fat diets	miRNAs	e.g., miR-10 b, miR-219-1-3P, miR-376a, miR-1897-5p, 146b, 29a, and 200c	Fontelles CC et al. (7)
C57BL/6 mice	Low-protein diet	DNA methylation	e.g., *Ano8, Hmga1*, and *Gnas*	da Cruz RS et al. (40)
		sncRNAs	e.g., miR-10b, miR-10a, let-7c, let-7d, tRNA-SeC-TCA, tRNA-ACG, and tRNA-Ile-TAT	
C57BL/6 mice	DDT	miRNAs	e.g., miR-10b, miR-205-5p, miR-204, miR-3535, miR-182, and let-7e	da Cruz RS et al. (41)
**SPERM EPIGENETIC ALTERATIONS ASSOCIATED WITH OTHER DISORDERS IN OFFSPRING**				
C57/Bl6:129S6/SvEvTac hybrid mice	Chronic stress	sncRNAs	e.g., miR-29c, miR-30a, miR-30c, miR-32, miR-193-5p, miR-204, miR-375, miR-532-3p, and miR-698	Rodgers AB MC et al. (59)
C57BL/6 mice	Restraint stress	DNA methylation	*Sfmbt2*	Ling Wu et al. (71)
FVB/NJ	Low-protein diet	sncRNAs	e.g., tRNA-Gly-CCC, tRNA-Gly-TCC, tRNA-Gly-GCC, tRF-Lys-CTT, tRF-His-GTG, and let-7	Sharma U CC et al. (72)
C57BL/6J	High-fat diet	sncRNAs	e.g., miR-10a, miR-10b, tRNA-Glu, tRNA-Gly, and tRNA-Val	Chen Q YM et al. (73)
**Paternally induced placenta effects**			**Molecular alteration**	
C57BL/6	Low protein diet	Gene expression	Nutrient transporters: *Atp2b1, Slc38a2, Slc2a1, and Slc2a4*; DNA methyltransferase 1 and 3L (*Dnmt1* and *Dnmt3L*) Paternally imprinted: *Mest* and *Snrpn*	Watkins AJ SS et al. (74)
C57BL/6	High-fat diet	Gene expression	*Cxcr4* in both gender, and *Ppar-alfa* and *Casp12* in male placentas only	Binder NK et al. (75)

More recent studies show that small non-coding RNAs, which are abundant in sperm (5, 76), play a more critical role (59, 72, 73). Several published reports demonstrated that the small RNA load in paternal sperm is modulated by environmental and lifestyle factors and can transmit environmentally induced phenotypes to the offspring (5, 55, 72, 73). Some of those studies implicated specific classes of small RNAs. For instance, miRNAs overexpressed in sperm of males exposed to different factors can replicate the effect of specific paternal exposures in offspring when injected into normal embryos (5, 59, 77). Other studies showed that tRNA fragments (tRF) (72, 73), the major small RNA subtype in mature sperm, also play a role in the transmission of disease phenotypes from fathers to offspring. Another recent report identified modifications (e.g., 5-methylcytosine) in sperm tRFs that increase their stability and could help explain how environmentally induced changes in tRFs composition in sperm occurs (78). It has been proposed that sperm small RNAs are delivered to the oocyte and modulate the transcriptome during the first few cell divisions, setting a signaling cascade that can impact embryonic development, but the details are still under investigation (76, 79).

The sperm non-coding RNA load and the abundance of specific RNA subtypes changes as the sperm travels through the male reproductive tract (72). It is well-established that the mature sperm is transcriptionally inactive, raising the question of how these cells acquire a non-coding RNA load in response to environmental insults (80). This issue has been addressed in elegant studies showing that the small non-coding RNA cargo in sperm is acquired through its interaction with extracellular vesicles produced by caput epididymis epithelial cells (72, 81, 82). Importantly, these studies show that the RNA content in the epididymis extracellular vesicles is modulated by environmental factors, although the exact mechanisms are still being investigated. In addition to molecular alterations present in the sperm cell itself, a recent study suggests that molecular signals in seminal fluid can also program phenotypes in offspring (83).

A variety of nutritional and environmental exposures can program the sperm epigenome, particularly sperm non-coding RNAs (5, 55, 59, 70). In agreement with that, we found that all paternal dietary and environmental factors that promoted with increased breast cancer development in offspring also led to alterations in sperm small non-coding RNA. We showed that an obesity-inducing diet altered the expression of miRNAs in sperm (6). Interestingly, we also detected differences in sperm miRNAs when males were fed diets with two different types of fat (animal or vegetable origin) with a total of 89 miRNAs differentially expressed between the two groups (7). In more recent studies, we found that a low protein diet or oral consumption of the pesticide DDT altered the distribution and content of the major small RNA species detected, including microRNAs and tRFs in paternal sperm (40, 41). More importantly, we showed that embryonic injections of miRNAs altered by those exposures can replicate the cancer phenotypes and play a functional role in the epigenetic transmission of breast cancer predisposition (41) as shown for other diseases (59).

### Other Possible Mechanisms

While it is increasingly evident that environmental and nutritional insults modulate the sperm epigenome in animal models and humans (70, 84), how those changes affect embryonic and fetal development is less clear. Some studies suggest that sperm RNAs can directly modulate gene expression in embryonic tissues and developing fetus (72). Other studies, however, indicate that another paternally induced mechanism of epigenetic inheritance worth exploring is changes in placental development (Table 2).

In eutherian mammals, such as human and rodents, the placenta connects the fetus to the mother and provides the nutrient and gas exchanges necessary for the proper fetal development (85). The placenta arises from the trophectoderm, the structure forming the outer cell layer of the embryo in the blastocyst stage (85). Improper placental development disrupts passage of nutrients and oxygen to the fetus and has been proven to have implications of future disease for the offspring, such as hypertension, type 2 diabetes, and cancer (86). Many studies have documented the effects of maternal experiences in pregnancy on placenta development and function (86, 87). Yet, despite the growing number of studies showing the importance of pre-conception paternal environmental exposures on offspring's health, little is known about the impact that pre-conception paternal environmental exposures can have on placenta formation, although some indication that this is the case exists (74, 75).

Paternal effects on the placenta are potentially important as its development is driven mostly by the paternal genome and epigenome (88, 89). In fact, Wang et al. found that the expression of paternally imprinted genes predominates in placenta but not in the fetal tissues, implying a large paternally driven impact on the transcriptome of the placenta (90). Paternally expressed genes specifically enhance feto-placental growth and varying epigenetic markers drive adaptations in placental phenotype depending on environment conditions. The nutritional status of the father seems to play a role. In agreement with that, gene expression patterns were found to differ between placentas derived from offspring of obese and non-obese fathers, with alterations being more pronounced in placentas of males than in those of female offspring (75). Placentas from obese fathers' male offspring showed significantly decreased expression of *Ppara* and *Casp12* while female placentas did not show this difference in gene expression. However, paternal obesity significantly increased global DNA methylation levels in female placentas, which could explain differences in phenotypic changes faced by male and female offspring. Thus, the placenta effects could hold a potential answer into how poor paternal nutrition can modulate breast cancer risk and other diseases in offspring. We are currently exploring whether sperm non-coding RNAs play a role in placenta development and ultimately modulate disease phenotypes such as breast cancer in offspring.

## Conclusions, Unanswered Questions, and Challenges

It has long been known that maternal experiences in pregnancy can modulate offspring's breast cancer risk in human populations and animal models. This is not surprising given that maternal environmental insults could directly affect the developing fetus' mammary gland. However, the past decade showed that history of exposure in the parental generation could lead to multigenerational and transgenerational predisposition for breast cancer. The evidence stems primarily from animal models and replication of those findings in human cohorts is urgently needed to determine whether transgenerational inheritance of breast cancer also occurs in humans.

In recent years, animal models have also provided important experimental support for epigenetic inheritance and showed that pre-conception paternal factors can modulate breast cancer development in daughters through the germline. In humans, epidemiological studies have linked food supply in the grandparental generation to health outcomes in the grandchildren (50) and paternal age and ethnicity to breast cancer development (57). As previously discussed, it is possible that the link between birth weight and breast cancer in human cohorts could also reflect paternal factors that are associated with size at birth (6, 40, 65, 67).

While a growing number of studies have shown that ancestral dietary and environmental factors can lead to intergenerational and transgenerational epigenetic predisposition to breast cancer, there is still a lot that we need to learn for how this happens mechanistically. Questions that still require answers in this emerging field include: How does paternal germline epigenetic programming alter embryonic mammary tissue development? How is epigenetically induced information transmitted between generations? Do the same mechanisms uncovered in animal models apply to humans? Can germline alterations be reversed in order to manipulate disease phenotypes in offspring? In addition to local mammary tissue alterations, do systemic metabolic changes in offspring play a role in environmentally induced breast cancer risk? These and other questions have important implications and need to be addressed with a multidisciplinary effort that will require knowledge from fields such as developmental and mammary gland biology, cancer biology, and epidemiology.

It is also important to note that we have observed a striking overlap between phenotypes induced by paternal and maternal dietary exposures on both mammary gland morphology and breast cancer development, suggesting that a common mechanism may exist. We have also observed consistent systemic metabolic changes and mammary tissue-specific alterations in energy-sensing pathways in offspring induced by paternal diet paradigms. The consequences of those alterations for mammary cancer development also need to be further explored.

The research summarized in this review challenges the current view of how breast cancer predisposition is transmitted between generations. If confirmed in humans, this research field could lead to a better understanding of non-genetic transmission of breast and other cancers and what drives tumor growth in a subset of women and how we can stop it. It could also lead to the development of population-based preventive strategies to reduce breast cancer incidence, including epigenetic markers to identify women at risk. This will be a daunting task though, as population studies to identify epigenetic markers of ancestral exposure will likely take decades, given the temporal distance between the parental exposure and breast cancer onset in daughters. Another challenge to overcome and confirm findings from animal models in humans is the difficulty in ascertaining exposures in parents retrospectively, although data from our lab and others (40, 41, 55, 61, 72) suggest that different environmental insults alter the same non-coding RNAs in the male germline and could presumably be used as a marker of exposure if confirmed in humans. Studies on epigenetic inheritance of breast cancer in humans will also need to take into account the possible interaction between ancestral exposures with lifestyle and environmental factors after birth. The good news is that there are existing multigenerational human cohorts that could be useful to study intergenerational and transgenerational breast cancer predisposition (13, 91).

Another possible strategy to reduce epigenetically induced predisposition to breast and other cancers in offspring would require interventions in the pre-conception window. Preliminary data from our lab and others suggest that environmentally induced effects on the male germline are transitory and can be reversed if the environmental insult is removed (41, 84). Implementation of such measures in the human setting would require specific medical recommendations to men of reproductive age akin to those already in place for women undergoing pre-conception counseling.

## Author Contributions

SA conceived and wrote the review. RC oversaw the literature research, and wrote and edited the manuscript. EC, MS, and JB contributed to the literature research, helped to write, and edit the review. All authors contributed to the article and approved the submitted version.

## Conflict of Interest

The authors declare that the research was conducted in the absence of any commercial or financial relationships that could be construed as a potential conflict of interest.

## Abbreviations

BRCA1: breast cancer 1 gene
BRCA2: breast cancer 2 gene
DDT: dichlorodiphenyltrichloroethane
DES: diethylstilbestrol
DOHaD: developmental origins of health and disease
EE2: ethinylestradiol
F0: parental generation
F1: first filial generation
F2: second filial generation
F3: third filial generation
N-3 PUFAs: omega-3 polyunsaturated fatty acids
N-6 PUFAs: omega-6 polyunsaturated fatty acids
sncRNAs: small non-coding RNAs
tRF: tRNA fragments.
